# Object detection in motion management scenarios based on deep learning

**DOI:** 10.1371/journal.pone.0315130

**Published:** 2025-01-03

**Authors:** Baocheng Pei, Yanan Sun, Yebiao Fu, Ting Ren

**Affiliations:** 1 School of Physical Education, Jinjiang College, Sichuan University, Chengdu, Sichuan Province, People’s Republic of China; 2 School of Physical Education, Nanning College for Vocational Technology, Nanning, Guangxi Province, People’s Republic of China; 3 College of Aviation Security, Civil Aviation Flight University of China, Beijing, People’s Republic of China; Ningbo University, CHINA

## Abstract

In athletes’ competitions and daily training, in order to further strengthen the athletes’ sports level, it is usually necessary to analyze the athletes’ sports actions at a specific moment, in which it is especially important to quickly and accurately identify the categories and positions of the athletes, sports equipment, field boundaries and other targets in the sports scene. However, the existing detection methods failed to achieve better detection results, and the analysis found that the reasons for this phenomenon mainly lie in the loss of temporal information, multi-targeting, target overlap, and coupling of regression and classification tasks, which makes it more difficult for these network models to adapt to the detection task in this scenario. Based on this, we propose for the first time a supervised object detection method for scenarios in the field of motion management. The main contributions of this method include: designing a TSM module that combines temporal offset operation and spatial convolution operation to enhance the network structure’s ability to capture temporal information in the motion scene; designing a deformable attention mechanism that enhances the feature extraction capability of individual target actions in the motion scene; designing a decoupling structure that decouples the regression task from the classification task; and using the above approach for object detection in motion management scenarios. The accuracy of target detection in this scenario is greatly. To evaluate the effectiveness of our designed network and proposed methodology, we conduct experiments on open-source datasets. The final comparison experiment shows that our proposed method outperforms all the other seven common target detection networks on the same dataset with a map_0.5 score of 92.298%. In the ablation experiments, the reduction of each module reduces the accuracy of detection. The two types of experiments prove that the proposed method is effective and can achieve better results when applied to motion management detection scenarios.

## Section 1: Introduction

Human movement behavior detection systems and related technologies have received widespread attention and application, and are gradually applied to the analysis and management of athletes’ competition and training [1]. However, in an athlete’s training or competition site, quickly analyzing the categories and locations of various targets in the field, including athletes, referees, sports equipment, sports field boundaries, etc., can effectively guide coaches’ training decisions for athletes [2]. Therefore, this part of the detection work is crucial for the subsequent analysis of the athlete’s movements. In recent years, the field of target detection has seen significant advancements due to the rapid development of deep learning technologies. These advancements have led to the widespread application of deep learning-based target detection methods in various scenarios. Within the realm of network structure research, a distinction has been made between two major types of target detection networks: two-stage and one-stage networks. Two-stage target detection networks, as proposed in articles [3–6], start by processing the input image through the first stage of the Region Proposal Network (RPN) to identify regions of interest. The second stage then refines these target predictions. The RPN serves to filter potential target regions and make preliminary predictions, which are further refined by the second stage to produce the final results. This method, while achieving higher accuracy, often incurs a trade-off with detection speed.

Two stage series target detection network is characterized by higher detection accuracy but slower detection rate, for this feature, one-stage series target detection network is proposed. One stage series target detection network learns all the generated anchors directly, which greatly accelerates the detection speed, but at the same time it learns the anchor features that are unfavorable to the network that cause the accuracy is not as good as the two stage series network of the same period. Current mainstream target detection networks have achieved good results on public datasets such as MS COCO [7] and PASCAL VOC [8], such as Dai et al. [9], who proposed a ResNeXt-101-DCN backbone with a Dynamic Head design that achieved an accuracy of 54.0% on MS COCO [7]; DETR with Improved De-Noising Anchor Boxes for End-to-End Object Detection network proposed by Zhang et al [10] by using a contrast method for denoising training, hybrid query selection for anchor initialization and a look forward twice scheme for regression box prediction. The method also achieved a good result of 48.3% accuracy at the 12th epoch and 51.0% accuracy at the 36th epoch on MS COCO [7] using ResNet-50 as backbone. The above mentioned method mainly focuses on streamlining the network structure to improve the processing efficiency of the algorithm and thus speed up the detection.

Two-stage and one-stage target detection networks have achieved good results in their respective experiments, but both types of current algorithms are specifically designed for public datasets. When these networks are applied to datasets from different domains, they frequently encounter challenges in generalization, leading to less satisfactory detection outcomes. This limitation stems from the diverse distribution of categories and regression features encountered in different scenarios, which can diminish the effectiveness of these networks in specialized settings, such as sports scenes. As a result, there is a growing demand for target detection algorithms that are specifically tailored for sports scenes. In response to this demand, numerous scholars have recently proposed modifications to existing networks to better suit their specific datasets. F In the realm of specialized target detection systems, recent developments showcase the adaptability and effectiveness of modern neural network architectures. For example, Xie et al. [11] engineered a bespoke two-branch convolutional neural network aimed at enhancing cloud detection capabilities critical for weather forecast analysis. This model was tailored specifically to differentiate various cloud types more accurately and swiftly, improving both the precision and reliability of meteorological predictions. Meanwhile, Zhu et al. [12] focused on refining the detection capabilities of the YOLOv5 architecture by incorporating a transformer module into the detection head. This modification has proven particularly effective for identifying and categorizing objects in complex street scenes captured by unmanned aerial vehicles (UAVs). The integration of transformer technology allows for better handling of the spatial complexities inherent in urban environments, thus enhancing the detection accuracy. Gao et al [13] proposed an ICCG-YOLO for detecting basketball referee signals. However, there is a lack of target networks for detecting targets in sports management scenarios.

On another front, Jing et al. [14] creatively merged the MobileNet [15] and U-Net [16] architectures to address the challenge of fabric defect detection. They introduced a median frequency balancing loss function to their hybrid model, which is particularly adept at emphasizing less frequent, yet crucial, anomalies in fabric textures. This approach not only increases the detection rate of subtle defects but also ensures a more balanced performance across various fabric types, thereby optimizing quality control in textile manufacturing. These instances illustrate the continuous advancements and specialization in neural network applications, demonstrating how tailored architectures and innovative loss functions can significantly improve the performance and applicability of detection systems in diverse fields. Despite these advancements, the development of an effective target detection network specifically for motion management scenarios remains an open challenge. In terms of motion object detection, Yang et al [17] proposed a motion target detection method based on background subtraction and updating. Yu et al [18] proposed a motion target detection algorithm based on YOLOV4, which directly applies the YOLOV4 algorithm to motion object detection. Zhang et al [19] proposed an improved ViBe motion target detection method to address the ghosting and shadowing problems of conventional visual background extraction algorithms in motion target detection. Most of these methods are traditional image processing methods proposed for the image characteristics of motion scenes, or deep learning-based target detection models in public scenes are directly applied to targets in motion scenes, which are often less effective. The unique requirements of motion management, such as the need to accurately track and identify dynamic targets in sports environments, demand innovative approaches that can adapt to the complexities of this domain. The main reason is that the data of the sports place has a large difference in characteristics with the data of other scenes, which mainly shows the following characteristics: 1) The target dataset on the sports field can be regarded as time series dataset with different frames, and the use of video detection often requires a huge amount of arithmetic power consumption, and when it is regarded as a single-frame picture for the detection of the target, then the time-domain relationship between different frames is missing, which will result in the misdetection of the data between the neighboring frames, leakage rate increases; 2) different degrees of target overlap, some ball targets such as table tennis balls and the body parts of the player, or rackets, tables, nets in the imaging picture there is positional overlap, to a certain degree of difficulty to the regression task; 3) multi-target phenomenon, there are more targets to be detected in the sports venues, and the differences between the targets are large, with the deepening of the network, the information of these features will be progressively more difficult to differentiation.

Meanwhile, the current computerized system of target detection networks widely adopts the detection strategy of deeply coupling the regression task with the classification task. The limitations of this computerized network modeling system become more pronounced in motion management scenes due to the inherent conflict between regression and classification tasks. This conflict affects the effectiveness of each task, impacting the detection of all targets. Consequently, current target detection methods struggle to achieve optimal results in sports venues, leading to a scarcity of impactful research outcomes. Therefore, in order to adapt to the temporal, target overlapping in different degrees, and multi-target problems exhibited by the target detection problem in sports management scenarios. At the same time, to fill the gap that there has been a lack of research on better target detection algorithms in sports management scenarios, we designed a supervised target detection network oriented to target detection in sports venues. During the design process, we proposed the use of the TSM to capture the temporal feature information between different frames. The Deformable Attention Transformer (DAT) is employed for feature extraction of multiple and overlapping targets. At the detection stage, a decoupled detection head is designed for target classification and regression, making the network suitable for scenarios with multiple and overlapping targets.

In summary, our contributions are mainly in the following areas:

- In order to solve the problem of high arithmetic requirement for video detection and loss of inter-frame time domain features in target detection, TSM module is added to the designed supervised target detection network to avoid the loss of time domain information.
- In order to solve the multi-target and target overlap problems in sports places, the DAT is used for feature extraction in the designed supervised target detection network.
- To address the coupling issue between regression and classification tasks in target detection networks, we have designed a decoupled head structure in our supervised target detection network. This structure introduces an attention mechanism into the detection head, which significantly mitigates the mutual conflict between regression and classification tasks. By separating these tasks, the decoupled head allows each to be optimized independently, leading to improved overall performance.

Our extensive comparative and ablation experiments clearly demonstrate the superior performance of our proposed method compared to several other approaches. It effectively tackles the diverse challenges encountered in sports scene target detection, thereby significantly improving detection accuracy in this domain. This paper is structured as follows: The first section provides an overview of the unique characteristics and challenges associated with target detection in sports management scenes, while also highlighting our contributions to addressing these challenges. The second section delves into various methods for target detection in motion management scenes and offers a classification of existing target detection approaches, shedding light on their strengths and limitations. The third section is dedicated to a detailed exposition of our proposed method, including the intricacies of the core network structure and the loss function employed. In the fourth section, we present a comprehensive comparison of our method with seven other state-of-the-art methods, followed by a series of ablation experiments designed to validate the effectiveness of our approach. The final section concludes the paper with a summary of our findings and a reflection on the implications of our work for the field of sports scene target detection.

## Section 2: Related work

In this section, relevant methods for motion scene target detection will be presented, including video-based detection and single-frame image-based detection, followed by anchor-based and anchor-free target detection methods.

### Section 2.1: Motion detection method

On the one hand. Current live motion detection for athletes can be broadly divided into two categories: video-based detection methods and single-frame image-based detection methods. First, most methods based on video detection, as discussed in articles [20–24], often rely on network structures that are tailored to specific datasets. For instance, Shi et al. [20] introduced a novel Trident-head structure to address the issue of inaccurate boundary prediction in existing methods. This structure models the action boundary by estimating the relative probability distribution around it, which enhances the regression accuracy of the borders. However, this approach also leads to an increase in the complexity of the detection head structure. method on the corresponding dataset is 69.3%, which is 2.5% higher than the previous best performance, but suffers from 74.6% latency. Wang et al. [21] proposed an encoder-based memory-enhanced appearance motion network (MAAM-Net). This network features an end-to-end structure for learning the appearance and motion features of a given input frame, a fusion memory module for connecting normal and abnormal actions, and a margin-based potential loss to reduce computational cost. Additionally, a patch-based stride convolution detection (PSCD) algorithm is employed to eliminate the degradation phenomenon. However, This method achieves a detection accuracy of 0.977 on the UCSD Ped2 dataset, but is only applicable to the detection of a few abnormal movements and may not be effective for distinguishing between a large number of normal actions. Cheng et al. [22] designed a real-time motion detection network (MDNet-LBPC) based on a single linear bottleneck and pooling compensation. In this network, the computationally intensive CNN block is replaced by a single linear bottleneck operator at the feature extraction stage to reduce computational costs. During the decoder stage, a pooling compensation mechanism is used to replenish lost feature information. Despite these improvements, the training process of this network is heavily dependent on the dataset, limiting its extendability to other datasets. Overall, while video-based detection methods offer promising results, they often face challenges related to network complexity, specificity to certain types of actions, and dataset dependency, which can hinder their applicability in diverse motion management scenarios. The method achieved 95.74% accuracy on a single action category. This type of method enriches the research on video detection, which is characterized by the design of different detection networks for the characteristics of the dataset, and the need to consider the dynamic change of the target in time, behavioral analysis, and the scenario of contextual information. Its advantage is that it can handle contextual information better, but the consumption of computational resources is high.

To address the issue of bloated algorithmic structures in this detection methods, researchers [25–27] have proposed single-frame image-based target detection methods. These methods approach motion detection as a target detection problem, aiming to streamline the process. Sun et al. [25] introduced an algorithm based on a two-stream convolutional neural network with multi-level feature fusion for gesture action detection. However, this method exhibits poor robustness and is less effective when applied to the detection of other motor actions. M et al. [26] proposed a deep learning motion recognition model called the Convolutional Pose Machine (CPM), which utilizes stacked hourglass networks for the recognition of muscle actions. Despite its effectiveness, the model is heavily dependent on hardware devices, limiting its scalability. Lin et al. [27] aimed to improve the accuracy of 2D human posture detection within the MediaPipe framework, which was found to be inaccurate. They employed a threshold correction method based on the speed of each joint, which includes correcting the Z-value of joint tilt angles, accurately adjusting the Z-value of different body postures, and addressing multi-frame jitter, hysteresis, and periodic noise problems caused by changes in joint velocity. This was achieved through one-ohm filtering and mean filtering of joint data. However, the algorithm design process involves substantial manual intervention in feature selection and is not a true end-to-end model. Overall, while single-frame image-based target detection methods offer a more streamlined approach to motion detection, they face challenges related to robustness, hardware dependency, and the need for manual intervention, which can limit their effectiveness and applicability in diverse motion management scenarios. As a result, such methods are generally not considered for time-continuous applications, and are usually highly accurate and relatively simple to implement, which has the advantage of dramatically reducing the consumption of computational resources, but also losing a large amount of temporal information, resulting in insufficient information obtained.

### Section 2.2: Anchor-based and anchor-free target detection methods

On the other hand, current computational mechanisms for target detection networks are predominantly divided into anchor-based and anchor-free mechanisms, which differ in their use of an anchor box during the prediction box generation process. The anchor box serves as a conventional a priori strategy for generating prediction boxes and has been widely adopted in the field since its introduction by Ren et al. [5]. Various classical networks [6, 28–33] have employed the anchor-based mechanism, which is known for its mature technology and high detection accuracy. In anchor-based target detection algorithms, determining hyperparameters such as the scale and aspect ratio of the anchor box, the number of generated anchor boxes, and the thresholds for intersection and overlap ratios is crucial. These parameters are typically based on the distribution of object dimensions and aspect ratios in the training data. The selection of a greater number of anchor boxes allows the network to learn more information, thereby enhancing its detection effectiveness. However, the reliance on a priori knowledge and the direct impact of hyperparameter selection on final accuracy result in a lack of generalization ability for this method. Moreover, anchor-based algorithms often generate significant computational redundancy due to the intensive calculations of intersection and overlap ratios between the anchor frame and the real bounding box during training. This computational burden is further exacerbated by the imbalance of positive and negative samples in the training data, which can lead to low detection accuracy for anomalous objects and multi-scale targets. Additionally, the need to screen a large number of generated anchor boxes at the detection end increases the complexity of the detection head. In some edge AI systems, this can create an overall latency bottleneck, particularly when transitioning from Neural Processing Units (NPUs) to Central Processing Units (CPUs) while making numerous predictions. In summary, while anchor-based target detection algorithms offer high accuracy and mature technology, they face challenges related to generalization ability, computational redundancy, and detection head complexity, which can impact their efficiency and applicability in certain scenarios.

The anchor-free mechanism emerged as a compelling alternative to the traditional anchor-based mechanism in target detection networks. Distinguished by its departure from a priori strategies, the anchor-free approach leverages direct feature information for generating prediction frames. This innovative concept was first introduced by Redmon et al. [34], and subsequent works [35–39] have proposed various classical structures that have been widely recognized and incorporated into different backbone networks. In anchor-free mechanisms, the focus is on predicting the object’s center, often accompanied by the computation of a soft centerness score to enhance the precision of detection. For bounding box prediction, these mechanisms typically estimate the distance from a pixel point to the four edges of the ground truth box, employing specific techniques to constrain the regression range. Prominent examples of this approach include CornerNet by Law et al. [36], ExtremeNet by Zhou et al. [37], and CenterNet by Duan et al. [39]. These methods are characterized by joint prediction strategies based on multiple keypoints, where bounding boxes are generated using combinations of keypoints such as the upper-left and lower-right corner points or the extreme points and center point. Further advancements in anchor-free mechanisms have been made by Tian et al. [38] with the proposal of FCOS, Zhou et al. [40] with CenterNet, and Liu et al. [41] with CSP. These methods are based on single-center point prediction techniques, where bounding boxes are formed using the centroid in conjunction with distances to the box edges or the centroid along with the width and height. Anchor-free mechanisms are renowned for their strong generalization ability, streamlined framework, and high accuracy in detecting targets of anomalous scales. This makes them particularly suitable for small target detection and contributes to a reduction in network parameters. However, despite these advantages, anchor-free mechanisms are not universally optimal for generalized target detection and may exhibit lower accuracy compared to anchor-based algorithms in certain scenarios. Therefore, current target detection networks still seek a balance between generalization ability, detection accuracy, and framework complexity.

## Section 3: Proposed method

**Fig 1:**
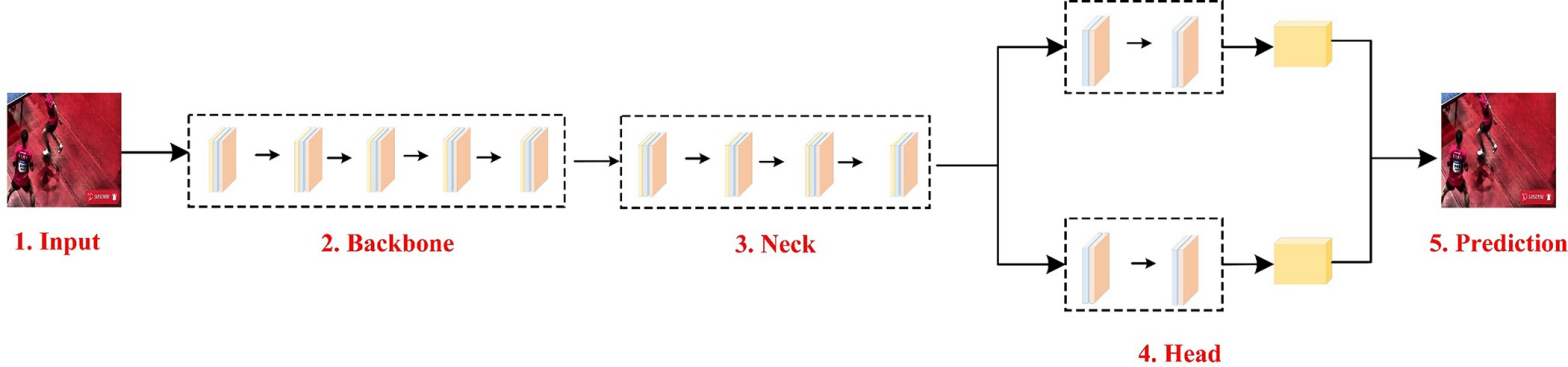
Overall network structure. This figure is the key to visualizing the network architecture, which includes individual connections and data flow paths.

In this section, we propose a supervised target detection network that is specifically tailored for target detection in sports scenes. As illustrated in Fig 1, our network architecture is designed with a focus on the high-speed detection requirements prevalent in athlete action recognition scenarios. To this end, we employ several components from the one-stage framework, which we have restructured to incorporate the TSM, DAT, and a decoupling head. These enhancements are primarily aimed at addressing the challenges of capturing temporal features, managing the overlap of targets, and resolving the coupling of regression and classification tasks in athlete action recognition, while also ensuring the real-time deployment capabilities of the subsequent engineering project. The overall structure of the network is divided into three main parts: Backbone, Neck, and Head. The Backbone utilizes a Focus + CSP (Cross-Stage Partial) structure, which provides a robust foundation for feature extraction. Neck adopts the FPN+PAN structure to better utilize the feature information extracted by Backbone, and embeds temporal features targeting athletes in action recognition, in addition to the deformable attention mechanism. This design enhances the network’s ability to capture the temporal and spatial relationships inherent in athlete action recognition scenarios. The Head of the network features our specially designed decoupled head structure, which comprises three detector heads. Each detector head is divided into a classification subhead and a regression subhead. This decoupled structure significantly improves the detection performance compared to traditional coupled structure detector heads across various types of target instances. By optimizing each component of the network for the specific demands of sports scene target detection, our proposed architecture aims to deliver high accuracy and speed in recognizing and tracking athlete actions. In subsequent chapters, we will detail the specifics of each innovative module.

### Section 3.1: TSM module

The TSM (Temporal Shift Module) module was originally designed to address the shortcomings of traditional Convolutional Neural Networks (CNNs) in processing temporal data, i.e., ignoring temporal information in time series. Traditional CNNs mainly focus on extracting spatial features while ignoring the evolution of temporal actions in the time dimension. The TSM module enables the model to effectively capture temporal information by introducing temporal shift operations. Specifically, the temporal offset operation offsets the input feature map along the time dimension, allowing features at each moment to interact with features at their neighboring moments. This interaction can help the model better understand the action change and timing relationship, thus improving the accuracy of action recognition.

**Fig 2:**
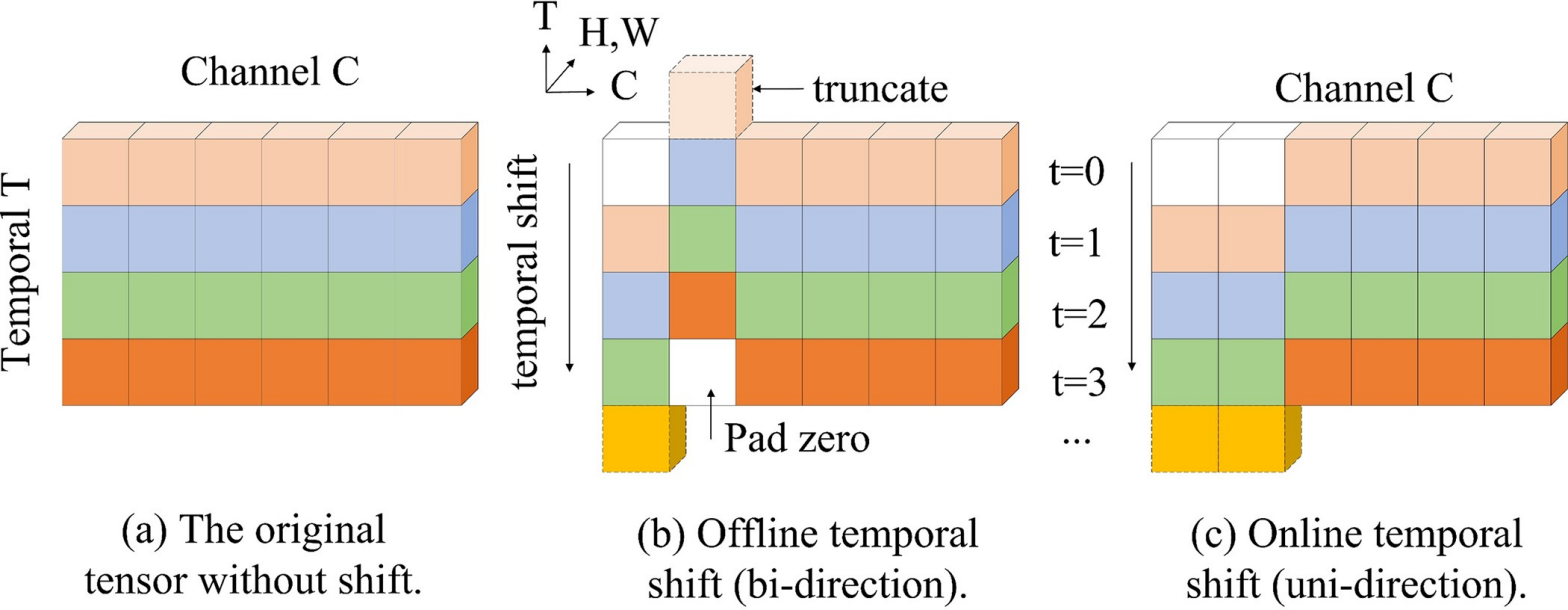
TSM is a specialized component primarily used in video processing networks to efficiently process temporal information within video frames. **TSM module.** Modules are designed to enhance the ability of convolutional neural networks to capture dynamic temporal features without significantly increasing computational cost or model complexity.

The TSM module typically consists of two key components: the temporal offset operation and the spatial convolution operation. The time offset operation is the core of the TSM module, which achieves the modeling of temporal information by offsetting the input feature map along the time dimension. Specifically, the time offset operation divides the input feature map into multiple time segments and applies a different degree of time offset to each time segment to realize the intersection of temporal features. The spatial convolution operation, on the other hand, is responsible for processing the spatial dimension of the feature map to extract spatial information and patterns. In order to make the designed network structure more accurate in capturing the close relationship between the before and after temporal actions in the recognition of athletes’ action behaviors, so as to improve the accuracy of target recognition in sports management scenarios. We added a time-shifted module (TSM) to the network structure, the principle structure of which is shown in Fig 2. The TSM captures the interrelationships between the front and back frames of the athlete’s consecutive behavioral actions by extracting the time-dimension channels in the network structure, and it enhances the overall network structure’s performance in the recognition of athletes’ behavioral actions by guiding the feature extraction task in the next moment using the feature information of the previous moment, and at the same time, calibrating the feature information in the previous moment using the feature information of the next moment. The TSM uses the feature information of the previous moment to guide the feature extraction task of the next moment, and uses the feature information of the next moment to calibrate the feature information of the previous moment to enhance the feature extraction ability of the overall network structure in the time dimension, so as to improve the accuracy of the athlete’s behavior recognition.

Traditional 2D CNNs operate independently in dimension T and, therefore, do not affect temporal modeling (Fig 2(A)). In contrast, TSM moves some channels forward and backward in the temporal dimension, and the information of neighboring frames will be mixed with the current frame, and the temporal information will be richer (Fig 2(B)). However, for real-time online inference, future frames cannot be moved to the present, so unidirectional TSM (Fig 2(C)) is used to perform online inference. During inference, the first 1/8 of the features are retained for each frame and cached. In the next frame, the first 1/8 of the current feature map is replaced by the cached features. The current 7/8 features are combined with the previous 1/8 features to produce the next layer, and then the process is repeated. The TSM module plays an important role in action recognition tasks, and has a wide range of applications, especially in athlete action recognition. In the next experiments, we will verify the effect of the TSM module on improving the performance of athlete action recognition, and analyze and discuss it more deeply to further reveal its working mechanism and advantages. The TSM module is designed for temporal information learning in motion target detection. Meanwhile, to further enhance the feature extraction capability for motion target detection, a variability attention mechanism is proposed in Section 3.2.

### Section 3.2: Deformable attention transformer

Existing target detection networks typically employ coded structures for image feature extraction. However, when applied to athlete action recognition, these networks often encounter several challenges. One significant issue is the information loss caused by the down-sampling technique used in convolutional structures. Additionally, the attention shifting mechanism of transformers leads to a slow expansion of the receptive field, which can hinder the network’s ability to effectively model large objects, such as those encountered in athlete action recognition. To address these limitations, there is a growing need to rely on the sparse attention of data to flexibly model relevant features. This requirement has led to the development of the deformation mechanism, which was first introduced by the Deformable Convolutional Networks (DCN). Building on this concept, this paper introduces the DAT mechanism specifically for athlete action recognition. The DAT mechanism aims to enhance the network’s ability to capture and model the dynamic and complex movements of athletes by providing a more flexible and adaptive approach to feature extraction. By incorporating the DAT mechanism into the target detection network, we seek to improve the accuracy and robustness of athlete action recognition, enabling the network to better handle the unique challenges presented by this application domain.

In our approach, we aim to efficiently model the relationships between tokens using the DAT. This is achieved by focusing on important regions within the feature map, which are identified by multiple sets of deformable sampling points. These sampling points are learned from queries through an offset network, allowing for adaptive selection of relevant features based on the input data. To process the identified focus regions, we employ bilinear interpolation to sample the features from these regions. The sampled features are then input into a key and value projection, resulting in the generation of deformed keys and values. This deformation process enables the network to capture and emphasize the most relevant features for the task at hand. Subsequently, standard polytope attention is applied to query the sampled keys and aggregate features from the deformed values. This step allows for the effective integration of information from the selected focus regions, enhancing the overall representational power of the network. Moreover, the location of the deformed points provides a more robust relative positional bias, which is crucial for the learning of deformation attention. This positional bias helps the network to better understand the spatial relationships between different parts of the input data, further improving the accuracy of the model. In the following sections, we will delve deeper into the specifics of the deformation attention mechanism and its implementation within our proposed network architecture. By leveraging the DAT, we aim to achieve a more effective and efficient modeling of token relationships, particularly in the context of target detection in dynamic and complex scenes such as athlete action recognition.

The processing flow of DAT is as follows: Input: input feature map; Step 1: establish global grid reference points according to the feature map, the size of the grid is obtained from the input feature map based on the factor *r* down-sampling; Step 2: the values of the reference points are the linear 2D coord inates of the grid, and the values are normalized to between [-1,+1] according to the grid size normalize to between [-1,+1], (-1,-1) represents the top-left grid point, (+1,+1) represents the bottom-right grid point; Step 3: To obtain the offset for each reference point, the feature map is mapped through the mapping matrix *W*_*q* to get *q*, and then *q* is fed into a lightweight sub-network *θ*_*offset*_ to get the offset for each Q; Step 4: Add the reference point values obtained from Step 2 and the Offset obtained from Step 3 to get the deformed points of the deformed mesh reference points; Step 5: Perform feature sampling at the location of the deformed mesh reference points, use bilinear interpolation as the sampling function, take the sampled deformed features as Key and Value, and then go through the projection matrices *W*_*v* and *W*_*k* to get $\bar{v}$ and $\bar{k}$; Step 6: Apply relative positional bias coding R to *q*, *k*, *v* then go through the classical multi-head attention layer, and the output of the single layer is as follows: finally, the features of each head output are spliced together, and then go through *Wo* for projection to get the final output.


$$z^{\left(m\right)}=\sigma (\frac{q^{\left(m\right)}{\tilde{k}}^{(m)T}}{\sqrt{d}}+\varphi (\hat{B};R)){\tilde{v}}^{\left(m\right)}$$ (1)

Applying DAT to the task of athlete action recognition can further improve the spatio-temporal understanding of athlete action data. By combining the self-attention mechanism and the DAT of the Transformer model, the model is able to understand the action information in the frame sequence more comprehensively, thus improving the accuracy and robustness of the recognition. In our study, we embed the DAT into our model and combine it with the TSM module to improve the accuracy and robustness of athlete action recognition. By jointly considering spatio-temporal information, our model is able to better distinguish different actions and is more sensitive to temporal changes in actions, thus further improving the performance of action recognition. After temporal information learning and feature extraction, the detection of specific targets is inevitably involved, and in order to adapt to target detection in motion scenarios, a novel decoupling head based on the attention mechanism will be proposed in Section 3.3.

### Section 3.3: Decoupled head with attention mechanism

In the realm of target detection networks, the simultaneous optimization of classification and regression tasks often leads to conflicts. This is primarily because these tasks share the neural network’s weights, despite requiring distinct weight information. Balancing the learning preferences of these tasks during network optimization proves to be a significant challenge. This issue is also prevalent in athlete action recognition, a specialized subset of target detection. To mitigate these conflicts, researchers such as Song et al. [42] and Guo et al. [43] introduced the concept of feature decoupling. This approach involves isolating and extracting the specific features required for classification and regression tasks. However, this process may result in the loss of some information. Efforts to decouple tasks have been made in the detection heads of both one-stage and two-stage networks. Nevertheless, these approaches typically employ a unified loss function in the final stage, which optimizes both tasks simultaneously but fails to achieve true decoupling. In response to these challenges, we have reengineered the detection head to distinctly separate the classification and regression tasks. By doing so, we leverage the unique characteristics of each task, allowing for more targeted optimization. The detailed structure of this redesigned detection head is depicted in Fig 4. This innovative approach aims to enhance the network’s overall performance by resolving the inherent conflict between classification and regression tasks within target detection networks.

**Fig 3:**
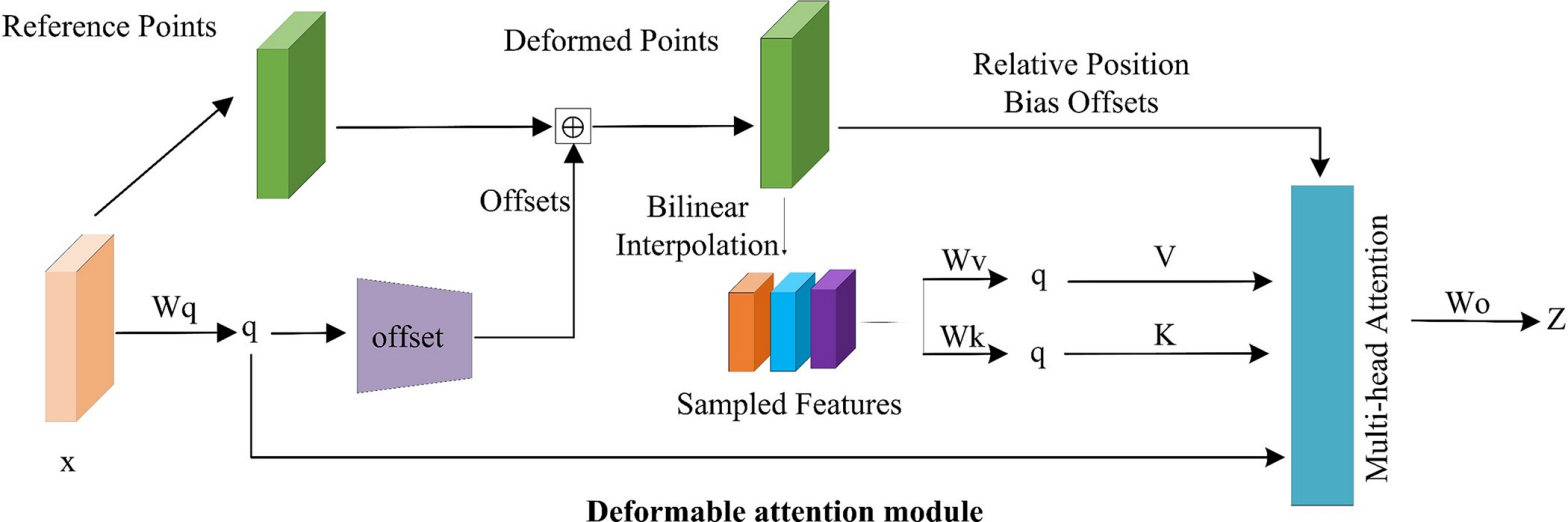
Deformable attention module.

**Fig 4:**
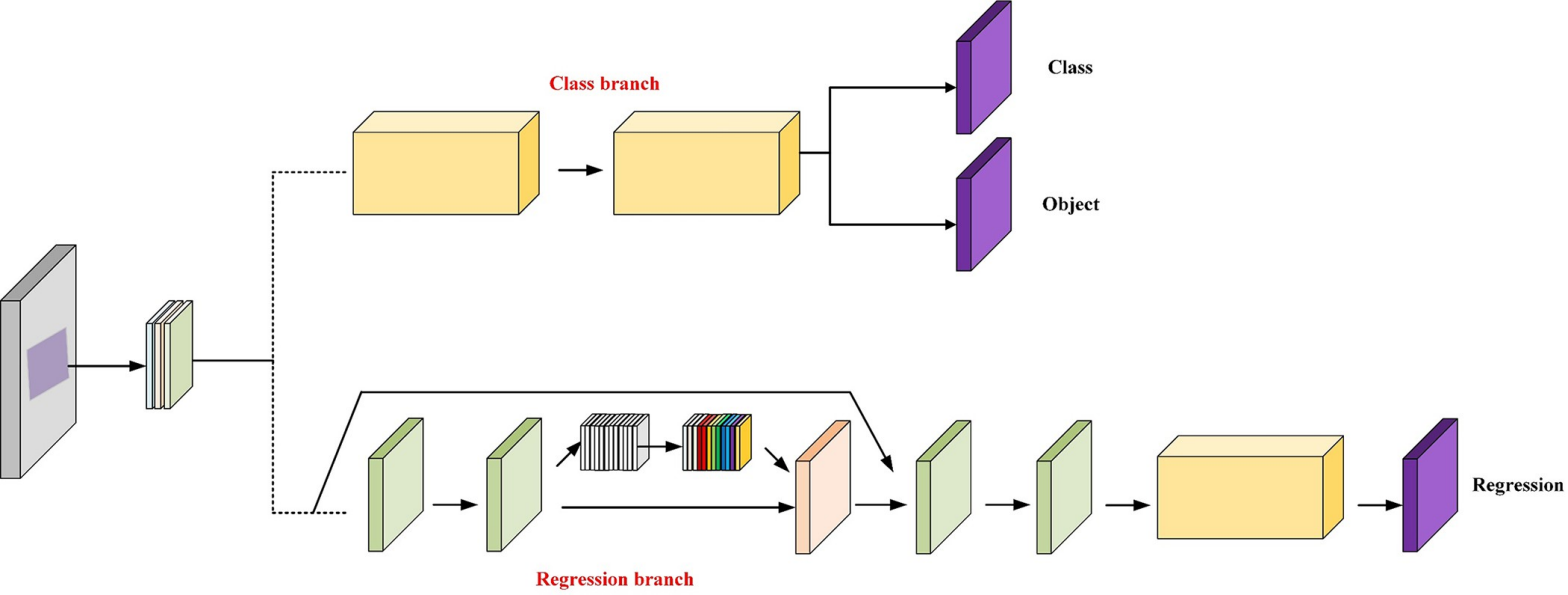
Decoupled header structure with attention mechanism. The decoupled header structure separates various tasks within the network into distinct components, allowing each component to specialize and optimize its operations. The integration of the attention mechanism further enhances the model’s ability to selectively focus on relevant features, improving accuracy and efficiency.

**Fig 5:**
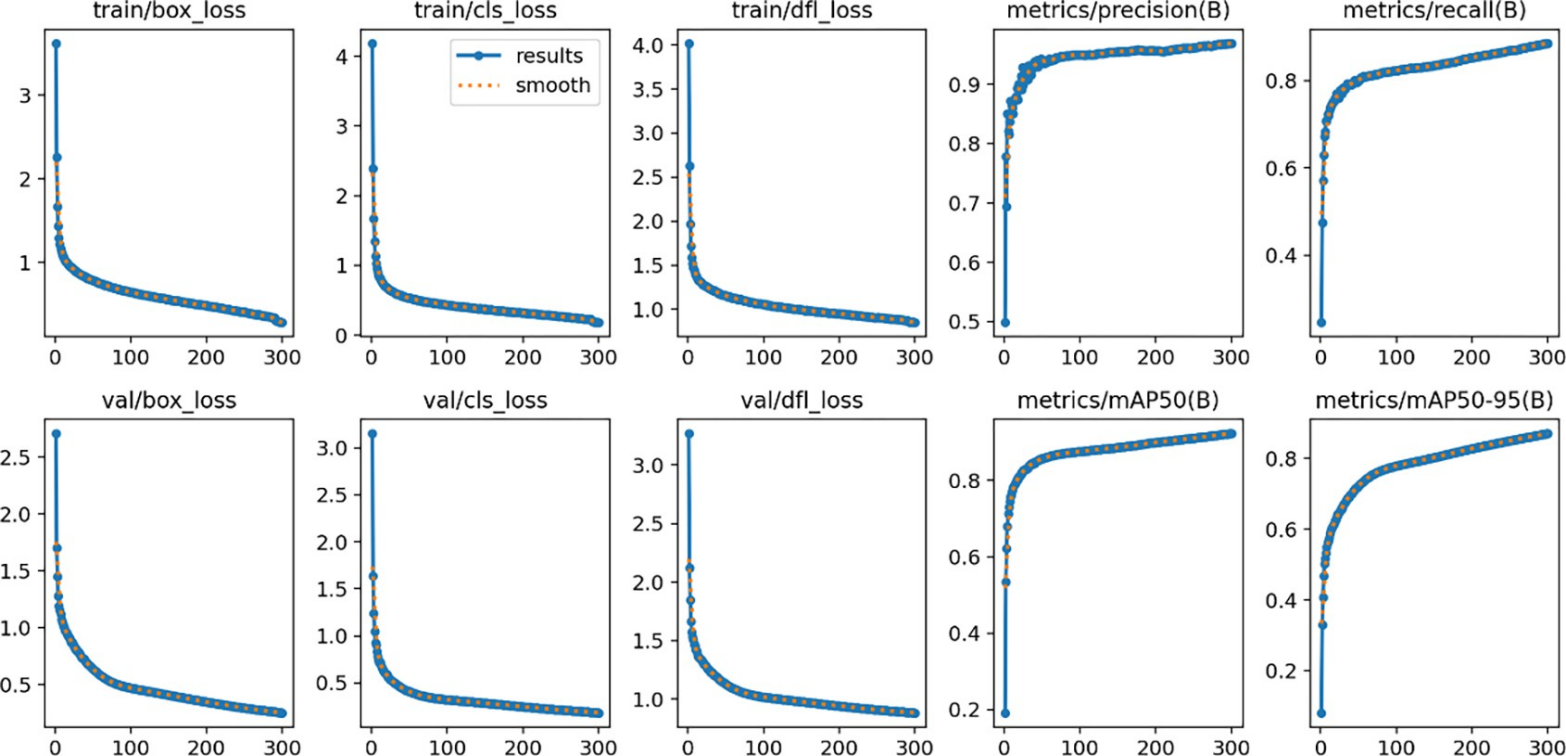
Training results for the network we designed. This graph provides a detailed visual analysis of the model’s performance over a series of training iterations, allowing for a comprehensive review of the model’s learning progress and results.

In our design, we adopt the detection head structure used in YOLOv4 and YOLOv5. This structure includes three scales of detection heads, each tailored to identify action targets of varying sizes. We have enhanced this design by decoupling each detection head into separate classification and regression task heads. This approach allows for independent optimization: the classification loss optimizes the classification head, while the regression loss fine-tunes the regression head, effectively separating the two tasks. The architecture of this decoupled head is depicted in Figs 3, 4. To further improve the decoupling effect, we have restructured the specific designs of the classification and regression subheads, tailoring them to their respective feature selection requirements. In the realm of classification networks, the final layer typically consists of a fully connected layer, which, as demonstrated by Wu et al. [44], offers superior classification performance compared to a convolutional layer. Therefore, in our decoupled head, we have replaced the original convolutional layer with a two-layer fully connected structure for the classification subhead. For the regression task, which concentrates on features pertinent to the target’s location, it is crucial to accurately convey location-related features to the detection head. Since a convolutional layer may not fully and accurately capture this location information, we have integrated a Coordinate Attention Module (CAM) before the convolutional layer. The CAM aids the regression subhead in precisely capturing location-related feature information by incorporating this data into channel attention. As illustrated in Fig 5, the addition of CAM enriches the location-related features obtained by the regression task subhead, validating CAM’s efficacy in boosting the regression task’s performance. Moreover, the confidence branch, associated with the classification task, shares the fully connected layer’s weight with the classification branch. This structural modification successfully decouples the regression and classification tasks, enhancing the overall effectiveness of our target detection network design. Next, the loss function based on supervised learning rules to measure the loss function between the true and predicted targets is presented in Section 3.4.

### Section 3.4: Loss function

In our athlete action recognition network, we employ a sophisticated approach that integrates multiple loss functions to finely tune and optimize the network’s performance. These loss functions include prediction frame to true frame regression loss, action classification loss, and confidence loss. Each of these components plays a critical role in training the network to accurately recognize and classify athlete actions from video data. This section will provide an in-depth discussion of how these loss functions are implemented and how they contribute to the overall effectiveness of the model.

### Section 3.4.1: Localization loss

In the regression task sub-detection head of an object detection model, the regression loss function plays a critical role. This loss is specifically designed to minimize the discrepancies between the predicted bounding box (prediction frame) and the ground truth bounding box (real frame). By applying this regression loss, the model is encouraged to adjust the coordinates of the predicted bounding box in such a way that it aligns more accurately with the actual bounding box of the object in the image. As a result, the prediction frame gradually becomes closer to the real frame, improving the model’s ability to accurately localize objects. For the action recognition characteristics of athletes, we use DFL Loss + *α*-IOU Loss, and the designed regression loss is shown below:

$$\alpha ‐IOU=1-{IOU}^{\alpha }+\frac{\rho^{2\alpha }(b,b^{gt})}{c^{2\alpha }}+{\left(\beta \upsilon \right)}^{\alpha }$$ (2)

$$DFL(S_{i},S_{i+1})=-((y_{i+1}-y)\log\left(S_{i}\right)+(y-y_{i})log(S_{i+1}))$$ (3)

$$where,\;\nu =\frac{4}{\pi^{2}}{\left(tanh\frac{w^{gt}}{h^{gt}}-tanh\frac{w}{h}\right)}^{2}$$ (4)

$$\beta =\frac{v}{(1-IOU)+v}$$ (5)

$$IOU=\frac{\left|b\cap b^{gt}\right|}{\left|b\cup b^{gt}\right|}$$ (6)

In Eq (2), *b* and *b*^*gt*^ denote the prediction frame and the true frame, respectively, *ρ*^2^(*b*, *b*^*gt*^) denotes the Euclidean distance between the prediction frame and the true frame, *c*^2^ denotes the square of the distance from the upper left corner to the lower right corner or from the lower left corner to the upper right corner between the prediction frame and the true frame, and *α* is a hyperparameter. In Eq (4), *w* and *h* denote the width and height of the predicted frame, and *w*^*gt*^ and *h*^*gt*^ denote the width and height of the real frame, respectively.

### Section 3.4.2: Classification loss and confidence loss

In the target detection task, the classification loss function serves as a critical guide for the learning process of the classification aspect of the model. This function is instrumental in training the model to correctly categorize objects within an image into predefined classes. The classification loss function is typically formulated based on the discrepancy between the predicted class probabilities and the actual, ground truth labels associated with the objects, the classification loss function can be expressed as:

$$L_{1}=-\frac{1}{n}\sum \left(y_{n}\times lnx_{n}+(1-y_{n})\times \ln\left(1-x_{n}\right)\right)$$ (7)

where n denotes the total number of categories, *x*_*n*_ is the predicted value of the current category, *y*_*n*_ is labeled 0 or 1, n takes the total number of action categories, and the incoming value *x*_*n*_ needs to be preceded by a sigmoid operation, the sigmoid formula is as follows:

$$S(x)=\frac{1}{1+e^{-x}}$$ (8)

## Section 4: Experiment

In this section, we conduct a comprehensive comparison between the method proposed in this paper and current state-of-the-art target detection algorithms. These algorithms encompass both two-stage and one-stage target detection approaches. For this comparison, we utilize a motion management dataset available on the AI community platform Hugging Face. To provide a thorough evaluation, we also perform a series of effective ablation experiments. These experiments are designed to isolate and assess the impact of individual components of our proposed method. By systematically removing or altering specific elements, we can determine their contribution to the overall performance of the model. The details of these comparisons and ablation experiments are meticulously documented in this section. Through this analysis, we aim to demonstrate the effectiveness and robustness of our proposed method in the context of motion management target detection, highlighting its advantages over existing state-of-the-art algorithms.

### Section 4.1: Datasets

The dataset in this chapter is from the open source dataset, totaling 14,132 sheets containing 7 common targets related to sports actions in 7 table tennis sports scenarios: ball, net, player, racket, scoreboard, table, and umpire. These targets include balls, nets, players, rackets, scoreboards, tables, and umpires, which are distributed across seven different types of sports fields. The resolution of the input image is 1392*1040 and the image preprocessing operations we take include mixup, mosaic. To facilitate a structured evaluation, the dataset is segmented into three subsets: the training set, the validation set, and the test set. Each subset is carefully curated to ensure a representative distribution of images, encompassing all seven target types across the various sports fields. This distribution is maintained consistently across the training, validation, and test sets to provide a comprehensive and balanced assessment of the model’s performance. The division of the dataset adheres to the ratio of 8:1:1, with the training set comprising 80% of the total images, and the validation and test sets each accounting for 10%. This allocation ensures that the model is trained on a substantial portion of the dataset while reserving a sufficient number of images for validation and testing purposes. By employing this dataset, we aim to rigorously evaluate the effectiveness of our proposed target detection method in recognizing and localizing sports-related targets within diverse sports field environments.

### Section 4.2: Comparisons with the state-of-the-art

To evaluate the effectiveness of the target detection method presented in this chapter, particularly in motion management scenarios, we conducted comparative experiments with state-of-the-art target detection algorithms. These algorithms include Faster R-CNN, RetinaNet, SSD, YOLOv3, YOLOv5, YOLOX, and YOLOv8. All experiments were conducted using the same dataset, referred to as dataset1. For the training process, each network algorithm was trained on an Nvidia GeForce RTX 3090 GPU. The algorithmic network framework utilized was PyTorch version 2.1.0. To ensure the fairness and comparability of the experiments, each set of experiments was trained for a consistent duration of 300 epochs, with a batch size of 16. The input images were uniformly resized to 640×640 pixels, and identical image preprocessing and data augmentation techniques were applied across all experiments. In addition patience = 50, workers = 8, optimizer set to auto, and learning rate set to 0.01. The training outcomes of the proposed method are depicted in Fig 5. A comparative analysis of the training results, juxtaposed with the outcomes of each network algorithm, is provided in Table 1. This comparative evaluation aims to highlight the advantages and performance of our proposed method in relation to the established state-of-the-art target detection algorithms in the context of motion management scenarios. During our experiments, we used the mAP_0.5, mAP_0.5:0.95, recall, and fps metrics for the comparison of various methods. These metrics are commonly used in target detection tasks to evaluate the performance and effectiveness of the model. mAP_0.5 and mAP_0.5:0.95 are commonly used target detection evaluation metrics to measure the accuracy of the model in predicting the target location, and they are computed separately for each category at the IoU (Intersection over Union, intersection-merger ratio) threshold of 0.5 for the Average Precision (AP, Average Precision), and then averaged the AP for all categories to get mAP_0.5, or calculated the AP for each category in the range of 0.5 to 0.95 and averaged it to get mAP_0.5:0.95. recall is used to measure the model’s ability to successfully find all the positive samples, i.e., how many samples predicted to be positive are true positive samples, a high recall means that the model is able to find more targets. FPS measures the speed of the model in processing frames per second, which directly affects the usefulness of the model in real-time applications.

**Table 1:** The comparison of the performance in our datasets with the state-of-the-art.

Method	map_0.5	map_0.5:0.95	recall	fps
Faster-rcnn	0.5529	0.4836	0.5137	6.1
retinanet	0.6683	0.4469	0.6932	5.3
SSD	0.5682	0.3805	0.5190	27.1
YOLOv3	0.6242	0.4087	0.5573	28.5
YOLOv5	0.8545	0.6443	0.8721	28
YOLOX	0.8272	0.5765	0.8921	31.6
YOLOv8	0.8726	0.8108	0.8615	85
Ours	0.92298	0.87038	0.88602	83

The experimental results presented above clearly demonstrate that the algorithm proposed in this chapter surpasses current state-of-the-art target detection algorithms in terms of both mAP metrics and recall metrics. In particular, our method shows a remarkable improvement of 35.478 percentage points in the mAP_0.5 metric compared to SSD. Additionally, it outperforms the best-performing YOLOX and YOLOv8 by 9.578 and 5.038 percentage points, respectively. These results indicate the strong applicability of our method in motion management recognition scenarios. Furthermore, the average frame rate of the proposed method reaches an impressive 83 frames per second, which is only slightly lower than YOLOv8 by 2 frames per second, ranking it second among all the evaluated methods. Notably, our method demonstrates superior speed compared to other algorithms such as Faster R-CNN, RetinaNet, SSD, YOLOv3, YOLOv5, and YOLOX. This balance of high accuracy and fast processing speed makes our proposed method particularly suitable for real-time applications in motion management scenarios.

The analysis of the experimental results presented above primarily demonstrates that the methods proposed in this chapter are more compatible with the target recognition scenarios of motion management species and exhibit better interpretability. There are three key points to be emphasized: (1) Temporal Sequence Characteristics: The targets in motion management scenarios are characterized by inter-frame temporal sequences. The networks used in the comparison experiments do not consider this feature information, leading to a higher number of false detections for targets of the same category. The Temporal Shift Module (TSM) designed in this paper, through the interaction of inter-temporal features, can better preserve temporal information, thereby significantly reducing the probability of false detection in motion management targets and improving overall detection accuracy. (2) Multi-Target and Overlapping Target Issues: In motion management scenes, there are challenges related to multi-target detection and target overlapping. While Faster R-CNN, RetinaNet, and SSD can address the multi-target problem by using various feature pyramid structures to enrich feature information during the feature extraction stage, they struggle with overlapping target detection. YOLOv3, YOLOv5, and YOLOX, despite adopting a multi-detection head structure, fail to accurately map captured features to specific action targets or background features due to the deepening of the network structure. In contrast, the DAT proposed in this paper effectively models the relationship between tokens under the guidance of important regions in the feature map. This approach enhances the extraction of feature information when targets overlap, resulting in a richer feature extraction process and ultimately improving overall detection precision. (3) In addressing the challenge of task decoupling in target detection systems, our research has identified that a significant degree of coupling between classification and regression tasks can lead to detrimental conflicts. These conflicts often degrade the overall performance of the detection network by interfering with the accurate assessment of object attributes and positional accuracy. To mitigate these issues, we introduced a novel concept focused on the decoupling of these tasks. Specifically, we developed a new type of detection head that incorporates an attention mechanism, aimed at minimizing the interdependence between classification and regression processes. The introduction of this attention-enhanced decoupling head is designed to strategically separate the tasks that traditionally compete for network resources. By doing so, it significantly diminishes the chances of errors such as false detections and missed targets. This approach helps in clearly distinguishing between the tasks of identifying what the objects are (classification) and determining where they are (regression). The reduced coupling ensures that each task can be optimized independently, thus enhancing the overall efficacy and reliability of the detection system. Our experimental results strongly support the effectiveness of this strategy, showing noticeable improvements in system performance by reducing task conflict. This development not only advances the state of technology in target detection networks but also sets the stage for more precise and reliable detection capabilities in a variety of applications. We anticipate that this innovation will provide a robust foundation for future advancements in the field and offer a valuable methodological shift that can be adopted across different types of detection systems. Overall, the proposed methods in this chapter address the unique challenges of motion management target recognition by effectively leveraging temporal information, handling multi-target and overlapping target issues, and reducing the coupling between classification and regression tasks.

**Fig 6:**
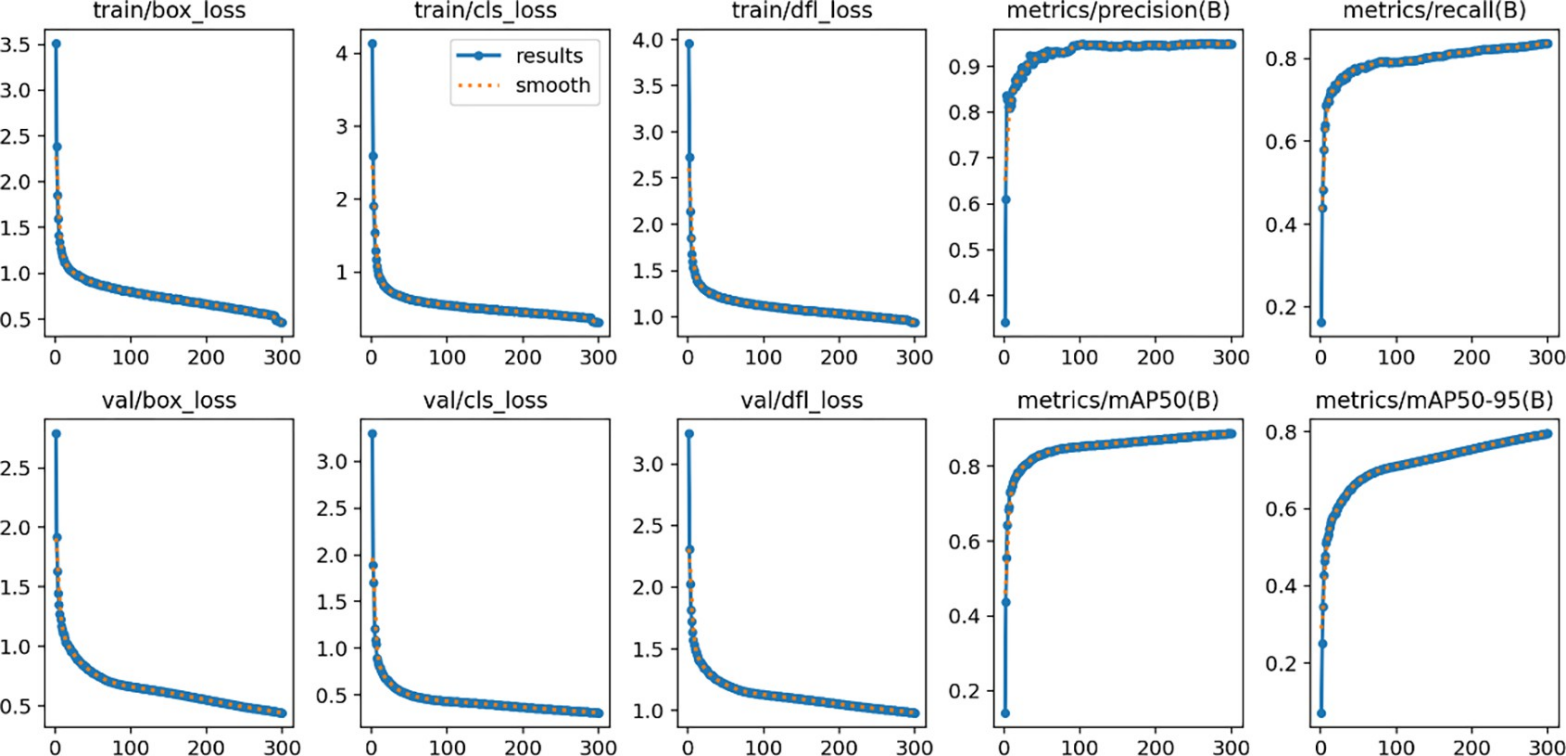
Training results of our network on dataset 2. This graph provides a detailed visual analysis of the model’s performance over a series of training iterations, allowing for a comprehensive review of the model’s learning progress and results.

Meanwhile, in order to verify the generalization performance of the model, we chose another set of basketball sports scene dataset dataset2, and experimented with the generalization performance of the model. The original dataset contains a total of five types of actions, including shooting, dribbling, free throws, defending, and fouling, with a total of 4,257 images. We benchmarked the performance of our network against several well-established networks in the field, with the results of these comparisons showcased in Fig 6. The performance metrics for each network, as detailed in Table 2, highlight the exceptional generalization capabilities of our proposed method. In the experiments conducted on dataset 2, our method achieved impressive metrics: an mAP_0.5 of 0.88266, an mAP_0.5:0.95 of 0.79539, and a recall of 0.3743. These results surpass those of the other seven competing methods across all three metrics, indicating that our method not only provides outstanding detection outcomes but also demonstrates robust generalization performance across diverse motion management scenarios. Encouraged by this success, we are now set to undertake a series of detailed ablation experiments. These experiments are aimed at thoroughly evaluating the contributions and effectiveness of the critical components integrated into our method, including the TSM, DAT, and the decoupling strategy employed in our detection head. Through these experiments, we seek to further validate the strength and adaptability of our approach in overcoming the challenges associated with motion management target detection, ensuring its applicability and reliability in real-world scenarios.

**Table 2:** The comparison of the performance in our dataset 2.

Method	map_0.5	map_0.5:0.95	recall
Faster-rcnn	0.7390	0.4564	0.6213
retinanet	0.827	0.451	0.514
SSD	0.7765	0.4376	0.7732
YOLOv3	0.7907	0.6231	0.7912
YOLOv5	0.8612	0.7765	0.8213
YOLOX	0.8512	0.7896	0.8187
YOLOv8	0.86782	0.8415	0.87034
Ours	0.88266	0.79539	0.83743

### Section 4.3: Ablation experiments

To evaluate the effectiveness of the method introduced in this paper, we carried out an extensive set of ablation experiments across all networks applying the proposed method. This approach ensured uniform conditions in terms of both the hardware used and the algorithmic framework. The central aim of these experiments was to analyze the impact and efficacy of critical components of our method, specifically the TSM, DAT, and the decoupled detection head that includes CAM. Therefore, in the ablation experiments, we tried the changes of the algorithm on three evaluation metrics, mAP_0.5, mAP_0.5:0.95, recall, when removing the TSM module and the DCN module, and when replacing the decoupling head with the detection head of YOLOV8, respectively. The findings from these studies are documented in Table 3, which provides a detailed examination of how each component influences key performance metrics, particularly mAP and recall. This table effectively contrasts the performance of networks with these components integrated versus their absence, thus illuminating their individual and collective contributions to the efficiency of the target detection system. In order to further verify that TSM and DCN still have an improved effect on other techniques for target detection in motion scenes, we tried to add TSM module and DCN module on YOLOV11 on dataset1, respectively. Table 4 shows the variation of the algorithm on the three evaluation metrics mAP_0.5, mAP_0.5:0.95, recall.

**Table 3:** The result of the ablation experiments.

Method	map_0.5	map_0.5:0.95	recall
Ours	0.92298	0.87038	0.88602
Without TSM	0.89296	0.85431	0.88265
Without DCN	0.91908	0.87012	0.86439
Replace decoupling head with YOLOv8 head	0.90975	0.86504	0.87248

**Table 4:** The result_2 of the ablation experiments.

method	map_0.5	map_0.5:0.95	recall
YOLOV11	0.90023	0.85134	0.84379
Add TSM	0.91274	0.86008	0.85236
Add DCN	0.90995	0.85823	0.85482

The data presented in Table 2 highlights the significant impact of our designed components on the performance of the target detection network. Specifically, the removal of the TSM results in a notable decrease in performance metrics: the mAP_0.5 value drops by 3.008 percentage points, the mAP_0.5:0.95 value decreases by 1.607 percentage points, and the recall index declines by 0.337 percentage points. This is mainly due to the fact that TSM draws on the shortcomings of traditional Convolutional Neural Networks (CNNs) when dealing with temporal data, and by introducing a temporal offset operation that offsets the input feature map along the temporal dimension, it allows the features at each moment to interact with their neighboring moments, which in turn improves the overall detection accuracy. Similarly, the elimination of the deformable attention mechanism we designed leads to a reduction in performance metrics: the mAP_0.5 value decreases by 0.39 percentage points, the mAP_0.5:0.95 value falls by 0.026 percentage points, and the recall metric drops by 2.163 percentage points. This is mainly due to the fact that Deformable Attention Transformer effectively models the relationship between features in different regions, and it provides a more robust relative positional bias through the location of the deformed points to facilitate the learning of the deformed attention, which is able to capture the complete feature information of each class of targets relative to the conventional feature extraction network, and thus improves the detection accuracy. Furthermore, replacing our designed decoupled head with the standard YOLOv8 head results in a decline in performance metrics: the mAP_0.5 value decreases by 1.323 percentage points, the mAP_0.5:0.95 value decreases by 0.534 percentage points, and the recall metric decreases by 1.354 percentage points. This can be attributed to the fact that our decoupled head effectively separates the regression and classification tasks of target detection, reducing the conflict between them compared to coupled structure detection heads, including YOLOv8, and thus enhancing the accuracy of target detection. Meanwhile, from the results in Table 4, it can be seen that after adding the TSM module we designed, its MAP value under the 0.5 threshold is improved by 1.251 percentage points, its MAP value under the average threshold from 0.5 to 0.95 is improved by 0.874 percentage points, and its RECORD metric is improved by 0.857 percentage points. This is mainly due to the fact that YOLOV11 is unable to obtain the timing information in the motion scene, and there is a lack of learning of this type of information in the network. Adding TSM can help the network structure to extract this type of feature information, which improves the overall detection accuracy. When adding the deformable attention mechanism we designed, its MAP value increased by 0.972 percentage points at the 0.5 threshold, its MAP value increased by 0.689 percentage points at the average threshold from 0.5 to 0.95, and its RECALL metric increased by 1.103 percentage points. This is mainly due to the fact that the deformable attention mechanism helps the feature extraction structure in the YOLOV11 network to extract richer feature information, which improves the detection accuracy. Overall, the results of these ablation experiments validate the effectiveness of our proposed methods and their potential for improved application in various target detection scenarios within motion management.

## Section 5: Conclusion

In this paper, for the gap of target detection methods in motion management scenarios, we start from the problem of timing loss, multi-targets, target overlap, and the widespread coupling of regression to classification tasks in the target detection network mechanism when using single-frame image target detection methods. A target detection method for motion management scenarios is proposed, and the main innovations of the method include a TSM module that captures timing information, a deformable attention mechanism that enriches feature information and avoids feature loss, and a decoupled detection head with a Coordinate Attention Module that decouples the regression and classification tasks. Finally, the results of comparative and ablation experiments are used to demonstrate that our proposed method is effective and is suitable for motion telomere scenarios with a significant improvement in detection on motion management datasets compared to other mainstream networks. We hope that this paper can bring some inspiration to the research work in industry or academia, and we also see that there is still some room for improvement of our network, and we will further improve our network for the unique problems in motion management scenarios, and will do further research on unsupervised detection. We also plan to collect a wider range of datasets including ball sports, track and field, and performance sports, and conduct more extensive experiments on these datasets as a way to validate the effectiveness of our model. Finally, we expect to further improve the efficiency of motion management target detection and make some contributions to the industry.
